# Supplementary material for: Identifying post-marital residence patterns in prehistory: A phylogenetic comparative analysis of dwelling size
Source: PLoS One. 2020 Feb 24;15(2):e0229363. doi: 10.1371/journal.pone.0229363 (PMC7039508; doi:10.1371/journal.pone.0229363)
Supplement: S1 File — (DOCX) [file pone.0229363.s001.docx]

# S1 Supporting Information

**Table A. Populations in the study sample, including those not included in the supertree.**

| **SOCIETY** | | **EA** | **Region** | **PMR** | **AHFA** | **MAT** | **AGR** | **SETTL** | **Ref. for AHFA** |
| --- | --- | --- | --- | --- | --- | --- | --- | --- | --- |
| Aleut | | Na9 | Subarctic America | 0 | 501 | D* | 1 | S | [1, p. 204] |
| Amhara | | Ca7 | Africa | 1 | 30 | I | 5 | S |  |
| Aranda | | Id1 | Australia | 0 | 3 | I* | 1 | M |  |
| Armenians | | Ci10 | West Asia | 1 | 60 | D | 5 | S |  |
| Aymara | | Sf2 | South America | 0 | 8 | D | 3 | S |  |
| Bemba | | Ac3 | Africa | 2 | 15 | I | 3 | S | [2, p. 100] |
| Blackfoot | | Ne12 | North America | 1 | 15 | I* | 1 | M |  |
| Burusho | | Ee2 | South Asia | 0 | 27 | I | 6 | S |  |
| Copper Eskimo | | Na3 | Subarctic America | 2 | 6 | I* | 1 | M | [3, p. 33] |
| Cagaba | | Sb2 | South America | 4 | 13 | I | 3 | S |  |
| Callinago | | Sb1 | Caribbean | 4 | 56 | D | 5 | S |  |
| Chiricahua | | Nh1 | North America | 3 | 5 | I* | 2 | M |  |
| Chukchee | | Ec3 | Siberia | 1 | 21 | I | 1 | M |  |
| Creek | | Ng3 | North America | 4 | 49 | I | 3 | S |  |
| Cuna | | Sa1 | Central America | 4 | 166 | D | 3 | S |  |
| Fang | | Ae3 | Africa | 0 | 14 | I | 3 | S |  |
| Ganda | | Ad7 | Africa | 2 | 56 | I* | 5 | S | [4, p. 103] |
| Garo | | Ei1 | South Asia | 4 | 57 | I | 3 | S |  |
| Gilbertese (Makin) | | If14 | Pacific | 1 | 30 | I | 4 | S |  |
| Gonds (Maria Gond) | | Eg3 | South Asia | 0 | 13 | I | 3 | S |  |
| Guarani | | Sj10 | South America | 0 | 18 | I* | 3 | S | [5, p. 82] |
| Hausa | | Cb26 | Africa | 0 | 11 | D | 6 | S |  |
| Havasupai | | Nd3 | North America | 0 | 19 | I* | 6 | S |  |
| Hupa | | Nb35 | North America | 1 | 31 | D | 1 | S | [6, pp. 13,16] |
| Iban | | Ib1 | Southeast Asia | 2 | 1083 | I | 3 | S | [7, p. 16, fig. 2] |
| Ifaluk | | If4 | Pacific | 2 | 11 | D | 4 | S | [8, p. 56] |
| Ifugao | | Ia3 | Southeast Asia | 2 | 7 | I | 6 | S | [9, p. 16] |
| Inca | | Sf1 | South America | 0 | 21 | D | 6 | S | [10, p. 166] |
| Japanese | | Ed5 | East Asia | 0 | 66 | D | 6 | S |  |
| Jivaro | | Se3 | South America | 2 | 158 | D | 3 | S | [11, p. 94] |
| Kanuri | | Cb19 | Africa | 0 | 11 | D | 5 | S |  |
| Kapauku | | Ie1 | Southeast Asia | 0 | 24 | D | 3 | S |  |
| Kaska | | Na4 | North America | 4 | 46 | D* | 1 | M | [12, pp. 59-61] |
| Kazak | | Eb1 | Central Asia | 0 | 15 | I | 2 | M |  |
| Khasi | | Ei8 | South Asia | 4 | 58 | D | 3 | S |  |
| Kol | | Eg8 | South Asia | 0 | 31 | D | 5 | S |  |
| Koreans | | Ed1 | East Asia | 0 | 59 | D | 6 | S |  |
| Lapps | | Cg4 | Europe | 1 | 13 | I | 1 | M |  |
| Lau Fijians | | Ih4 | Pacific | 0 | 41 | I | 4 | S |  |
| Makitare (Yekuana) | | Sc16 | South America | 4 | 398 | D | 3 | S | [13, p. 136] |
| Manus | | Ig9 | Southeast Asia | 0 | 67 | (I) | 2 | S |  |
| Maori | | Ij2 | New Zealand | 0 | 11 | I | 3 | S |  |
| Marquesan | | Ij3 | Pacific | 0 | 45 | I | 4 | S |  |
| Masai | | Aj2 | Africa | 0 | 14 | I* | 1 | M |  |
| Mataco | | Sh1 | South America | 3 | 4 | I* | 2 | M |  |
| Mbuti (Pygmies) | | Aa5 | Africa | 1 | 3 | I* | 1 | M |  |
| Miao | | Ed4 | East Asia | 0 | 28 | D | 6 | S |  |
| Miskito | | Sa9 | Central America | 4 | 192 | I | 3 | S |  |
| Mundurucu | | Sd1 | South America | 3 | 225 | I | 3 | S |  |
| Nicobarese | | Eh5 | Southeast Asia | 3 | 149 | I* | 4 | S |  |
| Nootka | | Nb11 | North America | 1 | 228 | D | 1 | S |  |
| Ojibwa (Pekangekum) | | Na34 | North America | 0 | 10 | D | 1 | S |  |
| Ona | | Sg3 | South America | 0 | 7 | I* | 1 | M |  |
| Paiute (Wadadokad) | | Nd22 | North America | 3 | 9 | I* | 1 | M |  |
| Papago | | Ni2 | North America | 0 | 18 | I | 6 | S |  |
| Rhade | | Ej10 | Southeast Asia | 4 | 79 | D | 3 | S |  |
| Rundi | | Ae8 | Africa | 0 | 28 | I* | 5 | S |  |
| Rwala | | Cj2 | West Asia | 0 | 46 | I | 1 | M |  |
| Santal | | Ef1 | South Asia | 0 | 17 | D | 6 | S |  |
| Semang | | Ej3 | Southeast Asia | 0 | 26 | I* | 1 | M |  |
| Serbs | | Ch1 | Europe | 0 | 42 | I | 5 | S |  |
| Seri | | Ni4 | North America | 0 | 6 | I* | 1 | M |  |
| Shavante | | Sj11 | South America | 4 | 45 | I* | 3 | M |  |
| Sinhalese | | Eh6 | South Asia | 1 | 56 | I | 6 | S |  |
| Siriono | | Se1 | South America | 4 | 139 | I* | 3 | M |  |
| Somali | | Ca2 | Africa | 0 | 9 | I* | 5 | M |  |
| Tanala | | Eh3 | Madagascar | 0 | 20 | I | 6 | S |  |
| Tapirape | | Sd2 | South America | 4 | 90 | I* | 3 | S |  |
| Tarahumara | | Ni1 | North America | 2 | 24 | D | 5 | S | [14, p. 120] |
| Tikopia | | Ii2 | Pacific | 0 | 25 | I* | 4 | S |  |
| Tiv | | Ah3 | Africa | 0 | 20 | D | 3 | S |  |
| Tlingit | | Nb22 | North America | 0 | 100 | D | 1 | S | [15, p. 125] |
| Toda | | Eg4 | South Asia | 0 | 15 | I* | 1 | S |  |
| Tonga (Plateau) | | Ac30 | Africa | 0 | 9 | I | 3 | S | [16, pp. 229-231] |
| Trukese | | If2 | Pacific | 4 | 28 | I | 4 | S |  |
| Tubatulabal | | Nc2 | North America | 1 | 29 | I* | 1 | M |  |
| Tucano | | Se12 | South America | 0 | 100 | (I) | (3) | (S) |  |
| Tupinamba | | Sj8 | South America | 3 | 1022 | I* | 3 | S |  |
| Tzeltal | | Sa2 | North America | 0 | 36 | (D)* | (3) | (S) |  |
| Warrau | | Sc1 | South America | 4 | 45 | I | 2 | S |  |
| Wolof | | Cb2 | Africa | 0 | 13 | I | 3 | S |  |
| Yahgan | | Sg1 | South America | 0 | 9 | I* | 1 | M |  |
| Yakut | | Ec2 | Siberia | 0 | 56 | D* | 2 | M | [17, p. 263] |
| Yanomamo | | Sd9 | South America | 0 | 784 | I | 3 | S | [18, p. 19] |
| Zulu | | Ab12 | Africa | 0 | 16 | I* | 3 | S | [19, p. 45] |
| Zuni | | Nh4 | North America | 4 | 104 | D | 6 | S |  |
|  | **NOT INCLUDED IN SUPERTREE** | | | | | | | | |
| Amahuaca | | Se8 | South America | 0 | 26 |  |  |  |  |
| Azande | | Ai3 | Africa | 0 | 9 |  |  |  |  |
| Bellacoola | | Nb9 | North America | 1 | 409 |  |  |  | [20, p. 257] |
| Hidatsa | | Ne15 | North America | 4 | 153 |  |  |  |  |
| Huron | | Ng1 | North America | 4 | 267 |  |  |  |  |
| Ila | | Ac1 | Africa | 0 | 19 |  |  |  | [21, p. 114] |
| Iroquois | | Ng10 | North America | 4 | 210 |  |  |  | [22, p. 181] |
| Klamath | | Nc8 | North America | 1 | 42 |  |  |  |  |
| Mandan | | Ne6 | North America | 4 | 136 |  |  |  |  |
| Maricopa | | Nh5 | North America | 0 | 29 |  |  |  |  |
| Nambicuara | | Si4 | South America | 1 | 16 |  |  |  |  |
| Omaha | | Nf3 | North America | 0 | 11 |  |  |  |  |
| Pawnee | | Nf6 | North America | 4 | 386 |  |  |  |  |
| Pukapukans | | Ii3 | Pacific | 0 | 20 |  |  |  |  |
| Ramcocamecra (Canela) | | Sj4 | South America | 4 | 32 |  |  |  | [18, p. 13] |
| Wintun (Wintu) | | Nc14 | North America | 1 | 13 |  |  |  | [23, p. 122] |
| **Legend**:  EA = ID in Ethnographic Atlas [24].  PMR = post-marital residence: *0 = patrilocal, virilocal, avunculocal and avuncu-virilocal; 1 = ambilocal, with a marked preponderance of virilocal practice; 2 = ambilocal, neolocal and avuncu-uxorilocal; 3 = ambilocal, with a marked preponderance of uxorilocal practice; and 4 = matrilocal and uxorilocal.* Data adapted from D-PLACE variable EA012.  AHFA = average house floor area (in m^2^). Data without reference are adapted from ref. [25].  MAT = wall material: I *= impermanent material; D = durable material. Label * means that walls are indistinguishable from roof or merging into the latter.* Data adapted from D-PLACE variable EA081 or EA083. *(I) or (D) = impermanent or durable material from another source than EA081 (see below).*  AGR = intensity of agriculture: *1 = no agriculture; 2 = casual agriculture; 3 = extensive or shifting agriculture; 4 = horticulture; 5 = intensive agriculture; and 6 = intensive irrigated agriculture.* Data adapted from D-PLACE variable EA028. *(3) = extensive or shifting agriculture* *from another source than EA028 (see below).*  SETTL = fixity of settlement: *M = mobile; S = sedentary.* Data adapted from D-PLACE variable EA030. *(S) = sedentary settlement from another source than EA030 (see below).* | | | | | | | | | |

## Changes to the original Porčić’s sample

Porčić [25] uses AHFA of 65.7 m^2^ for Kaska people from Divale’s study [26]. According to primary literature [12], this value corresponds to circular lodge of the Dease river Kaska. However, the same author also mentions Upper Liard Kaska, who build smaller conical lodges with AHFA of 29 m^2^. We therefore averaged both values into one, i.e., 46 m^2^. In reference [25], AHFA for Iroquois is an average of Ember’s [22] and Brown’s [18] values. In our sample, however, we prefer to use Ember’s value of 210 m^2^, since Brown’s code is for a single household cabin adopted after contact, not for traditional longhouse. Porčić [25] says he excluded Yakut due to conflicting reports of post-marital residence. In fact, the conflicting variable was AHFA – in Ember’s sample 56 ft^2^, while in Brown’s 56.3 m^2^. Since data in primary literature [17] are consistent with Brown’s code, we included Yakut back into the sample. Porčić [25] codes Tanala’s agriculture as “*not important*”, however, the code in *D-PLACE* [EA028] describes intensity of agriculture as “*Intensive irrigated*”.

## Codes different to original variables in D-PLACE

Tucano and Tzeltal are not scored for variables EA028, EA030 and EA081. In case of Tucano, data for related population Cubeo (Se5) in D-PLACE was used instead. Tzeltal traditionally practiced extensive or shifting agriculture [27], lived sedentarily and had durable house walls made of wattle-and-daub or plain tree trunks tied with vines [28]. Manus is not scored for variables EA081, but their wall material is not durable – house is thatched with sago-leaf thatch from ridge pole to floor [29].

## References

1. Veniaminov I. Zapiski ob ostravach Unalaškinskago otděla. Vol. 2. St. Petersburg: Tip. Imp. Rossijskoj Akad.; 1840.

2. Richards A. Land, labour and diet in Northern Rhodesia: an economic study of the Bemba tribe. London and New York: Oxford University Press; 1939.

3. Jenness D. The People of the Twilight. Chicago: University of Chicago Press; 1959.

4. Southwold M. The Ganda of Uganda. In: Gibbs JL, editor. Peoples of Africa. New York: Holt, Rinehart and Winston; 1965. p. 81-118.

5. Métraux A. The Guaraní. In: Steward JH, editor. Handbook of South American Indians, Vol 3, The Tropical Forest Tribes. Washington D.C.: Smithsonian Institution; 1948.

6. Goddard PE. Life and Culture of the Hupa. Berkley: The University Press; 1903.

7. Freeman D. The family system of the Iban of Borneo. In: Goody J, editor. The developmental cycle in domestic groups. Cambridge: Cambridge University Press; 1958. p. 15-52.

8. Senfft A. Die Karolineninseln Oleai und Lamutrik. Dr A Petermanns Mitteilungen aus Justus Perthes' geographischer Anstalt. 1905;51:53-7.

9. Burton RF. Philippine pagans: the autobiographies of three Ifugaos. London: George Routledge & Sons; 1938.

10. Cobo B. Historia del Nuevo mundo. Sevilla: Imp. de E. Rasco; 1893.

11. Karsten R. The Head-hunters of Western Amazonas: The Life and Culture of the Jibaro Indians of Eastern Ecuador and Peru. Helsingfors: Centraltryckeriet; 1935.

12. Honigmann JJ. The Kaska Indians: An Ethnographic Reconstruction. New Haven: Yale University Press; 1954.

13. Wilbert J. Survivors of Eldorado: Four Indian Cultures of South America. New York: Praeger Publishers; 1972.

14. Champion JR. A study in culture persistence: the Tarahumaras of northwestern Mexico. PhD Thesis, Columbia University. 1962.

15. Krause A. Die Tlinkit-Indianer. Jena: Hermann Costenoble; 1885.

16. Reynolds B. The material culture of the peoples of the Gwembe Valley. New York: Praeger; 1968.

17. Tokarev SA, Gurvich IS. The Yakuts. In: Levin MG, Potapov LP, editors. The Peoples of Siberia. Chicago: University of Chicago Press; 1964. p. 243-304.

18. Brown BM. Population estimation from floor area: A restudy of “Naroll’s Constant”. Behavior Science Research. 1987;21:1-49.

19. Krige EJ. The Social System of the Zulus. Pietermaritzburg: Shuter & Shooter; 1965.

20. Mackenzie A. Voyages from Montreal through the continent of North America to the frozen and Pacific oceans in 1789 and 1793, with an account of the rise and state of the fur trade. Vol 2. Toronto: The Courier Press; 1911.

21. Smith EW, Dale AM. The Ila-speaking peoples of Northern Rhodesia. Vol 1. London: MacMillan and Co.; 1920.

22. Ember M. An archaeological indicator of matrilocal versus patrilocal residence. American Antiquity. 1973;38(2):177-82.

23. Du Bois C. Wintu ethnography. Berkley: University of California Press; 1935.

24. Murdock GP. Ethnographic atlas: a summary. Ethnology. 1967;6:109-236.

25. Porčić M. House Floor Area as a Correlate of Marital Residence Pattern: A Logistic Regression Approach. Cross-Cultural Research. 2010;44(4):405-24.

26. Divale WT. Living floor area and marital residence: A replication. Behavior Science Research. 1977;12(2):109-15.

27. Turner PR. Intensive Agriculture among the Highland Tzeltals. Ethnology. 1977;16(2):167-74.

28. Villa Rojas A. Tzeltal. In: Vogt EZ, editor. Handbook of Middle American Indians Vol 7 and 8: Ethnology. Austin, Texas: University of Texas Press; 1969. p. 195-225.

29. Mead M. Kinship in the Admirality Islands. New York: American Museum of Natural History; 1934.
